# Resistance to neoplastic transformation of *ex-vivo* expanded human mesenchymal stromal cells after exposure to supramaximal physical and chemical stress

**DOI:** 10.18632/oncotarget.12678

**Published:** 2016-10-15

**Authors:** Antonella Conforti, Nadia Starc, Simone Biagini, Luigi Tomao, Angela Pitisci, Mattia Algeri, Pietro Sirleto, Antonio Novelli, Giulia Grisendi, Olivia Candini, Cintia Carella, Massimo Dominici, Franco Locatelli, Maria Ester Bernardo

**Affiliations:** ^1^ Department of Pediatric Hematology/Oncology, IRCCS Bambino Gesù Children's Hospital, Rome, Italy; ^2^ Department of System Medicine, University of Rome “Tor Vergata”, Rome, Italy; ^3^ Laboratory of Medical Genetics, IRCCS Bambino Gesù Children's Hospital, Rome, Italy; ^4^ Department of Medical and Surgical Sciences for Children & Adults, Division of Oncology, University-Hospital of Modena and Reggio Emilia, Modena, Italy; ^5^ Istituto Superiore di Sanità, Rome, Italy; ^6^ Department of Pediatrics, University of Pavia, Pavia, Italy; ^7^ Current address: San Raffaele-Telethon Institute for Gene Therapy, SR-TIGET, Pediatric Immunohematology, San Raffaele Scientific Institute, Milan, Italy

**Keywords:** mesenchymal stromal cells, ionizing radiation, starvation, malignant transformation, biosafety

## Abstract

The risk of malignant transformation of *ex-vivo* expanded human mesenchymal stromal cells (huMSCs) has been debated in the last years; however, the biosafety of these cells after exposure to supramaximal physical and chemical stress has never been systematically investigated.

We established an experimental *in vitro* model to induce supramaximal physical (ionizing radiation, IR) and chemical (starvation) stress on *ex-vivo* expanded bone marrow (BM)-derived huMSCs and investigated their propensity to undergo malignant transformation. To this aim, we examined MSC morphology, proliferative capacity, immune-phenotype, differentiation potential, immunomodulatory properties and genetic profile before and after stressor exposure. Furthermore, we investigated the cellular mechanisms underlying MSC response to stress. MSCs were isolated from 20 healthy BM donors and expanded in culture medium supplemented with 5% platelet lysate (PL) up to passage 2 (P2). At this stage, MSCs were exposed first to escalating doses of IR (30, 100, 200 Gy) and then to starvation culture conditions (1% PL).

With escalating doses of radiation, MSCs lost their typical spindle-shaped morphology, their growth rate markedly decreased and eventually stopped (at P4-P6) by reaching early senescence. Irradiated and starved MSCs maintained their typical immune-phenotype, ability to differentiate into adipocytes/osteoblasts and to inhibit mitogen-induced T-cell proliferation. The study of the genetic profile of irradiated/starved MSCs did not show any alteration. While the induction of supramaximal stress triggered production of ROS and activation of DNA damage response pathway via multiple mechanisms, our data indicate that irradiated/starved MSCs, although presenting altered morphology/growth rate, do not display increased propensity for malignant transformation.

## INTRODUCTION

Human mesenchymal stromal cells (MSCs) are multipotent cells that can be found in a variety of adult tissues, including bone marrow (BM) and adipose tissue (AT), besides fetal tissues such as fetal lung and blood [1, 2]. The ability to differentiate into mesoderm-derived cells, such as adipocytes, chondroblasts, and osteoblasts is considered as one of the minimal criteria to define MSCs [3, 4, 5, 6]. Within the BM, MSCs play a key role in sustaining hematopoietic stem cell functions and in regulating the production and maturation of hematopoietic progenitors, mainly through secretion of paracrine factors [6]. MSCs have also been shown to possess broad immune-regulatory properties through which they are able to influence both adaptive and innate immune responses [7, 8, 9]. Considering their fundamental role in immune regulation and in balancing tissue homeostasis, MSCs have been administered to patients, within experimental phase I-II clinical studies, with the aim of repairing tissues and blunting immune responses [10, 11, 12]. Based on these initial clinical findings, MSC anti-inflammatory therapy is being considered as a novel and potentially effective therapeutic option in the rapidly evolving field of Regenerative Medicine [13].

For the sake of patient safety, any clinical application of MSCs should take into account potential risks, such as ectopic tissue formation [14, 15] and tumor generation caused by malignant transformation of the cells during *ex-vivo* expansion [16]. After initial reports on the occurrence of neoplastic transformation in *ex-vivo* expanded huMSCs after long-term culture [16, 17, 18] this event has been subsequently described as uncommon, with an estimated frequency of <10^−9^ [19, 20, 21, 22]. Furthermore, neither ectopic tissue formation nor MSC-originating tumors have ever been reported so far in hundreds of patients treated with MSC therapy [23].

To expand the investigation on the biosafety profile of *ex-vivo* expanded MSCs, we have set-up an *in vitro* model aimed at testing their resistance to malignant transformation by inducing massive physical and chemical stress. For this purpose, we have employed ionizing radiation (IR) in combination with deprivation of nutrients as stressors during *ex-vivo* culture of BM-derived MSCs isolated from healthy donors (HD-MSCs) and expanded in the presence of a GMP-compliant culture medium which also includes platelet lysate (PL). Our results indicate that *ex-vivo* expanded huMSCs are resistant to these stressors and are not prone to undergo neoplastic transformation even after application of supramaximal doses of IR and starvation culture.

## RESULTS

### Characterization of stressed MSCs

**Figure 1:**
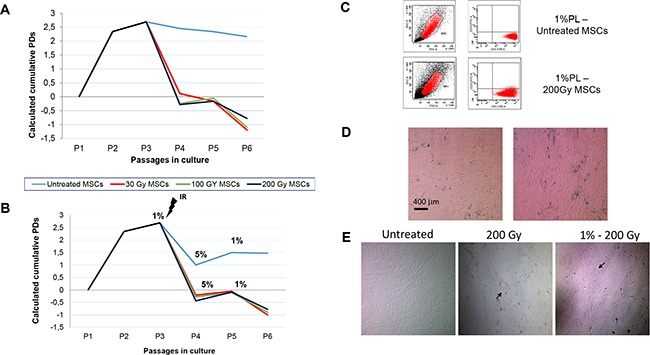
Calculated population doublings (PDs) from passage (P)1 to P6 of bone marrow (BM)-derived human mesenchymal stromal cells (huMSCs) exposed to ionizing radiation (IR) in the presence of a 5% platelet lysate (PL) additioned medium (A) and in starvation conditions (B) MSCs were irradiated with escalating doses (30, 100 and 200 Gy) at P2, and then plated with 5% or 1%PL. Starvation culture consisted of alternating 1% PL and 5% PL at each passage. Graphs show the mean of three different MSCs samples. After IR exposure, both 5% PL- and 1% PL-cultured MSCs show a marked decrease in their proliferation rate. **C.** Immunophenotype of untreated MSCs and 200 Gy irradiated MSCs from one representative sample, in starvation culture. Both untreated and stressed MSCs proved to be negative for CD33 and positive for CD13 surface markers. As shown in the morphological dot plot, MSCs became larger and looked exhausted after 200 Gy irradiation. **D.** ß-Galactosidase staining of MSCs from one representative healthy donor (HD) exposed to 30 Gy (left panel) or 200 Gy (right panel) IR and, then, to starvation culture. Senescence of the cells is indicated by the positivity for the staining. **E.** Morphology of culture-expanded MSCs obtained from one representative HD and exposed to stressors. *Left panel*: morphology of untreated MSCs; *central panel:* morphology of irradiated MSCs in the presence of 5% PL; *right panel:*morphology of irradiated MSCs in the presence of 1% PL. Magnification x5. Black arrows spotlight alterations in MSC morphology.

Although being exposed to increasing IR doses, MSCs still proved to be able to adhere to plastic surface, though they could be replated only for few more passages (up to P6). As far as the proliferative capacity is concerned, the proliferation rate of stressed MSCs was significantly lower in comparison to untreated MSCs, both in the presence of 5% PL and after nutrient deprivation. Indeed, irradiated MSCs, cultured in the presence of 5% PL, showed the following calculated cumulative PDs from P1 to P6: untreated MSCs = 11.98 (SD ±0.99); 30 Gy = 3.79 (SD±1.53); 100 Gy = 3.64 (SD ±1.53); 200 Gy = 3.81 (SD ±1.48; P<0.001 as compared with untreated MSCs for all IR doses; Figure 1A). No significant correlation was found between the doses of IR and the inhibitory effect on MSC growth rate (P>0.05). The calculated cumulative PDs of irradiated and starved MSCs were as follows: 1% untreated MSCs = 9.01 (SD ±0.96); 30 Gy = 3.77 (SD ±1.51); 100 Gy = 3.75 (SD ±1.51); 200 Gy = 3.73 (SD ±1.49; P<0.001 in comparison to untreated MSCs for all IR doses; Figure 1B).

MSC immunophenotype was evaluated by means of flow-cytometry after being irradiated and cultured in conditions of nutrient deprivation. Stressed MSCs showed the typical phenotype [6], which was not altered by the induced physical and chemical insults. In particular, as shown in Figure 1C, more than 98% of MSCs showed negativity for CD33 and positivity for CD13. Moreover, stressed MSCs proved to be negative for CD14, CD45, CD80 and HLA-DR, and positive for CD73, CD90, CD105 and HLA-I (data not shown).

A sharp decrease in MSC proliferation rate, progressing to cell-cycle arrest, and the positivity for β-Gal staining (Figure 1D) suggested an early replicative senescence (at P4-P6) of stressed MSCs, as expected after massive IR doses and nutrient deprivation. These observations are supported by the stress-related morphology alterations in MSCs undergoing IR and starvation culture. Furthermore, increasing IR dose treatment markedly altered MSC typical spindle-shape morphology, which also displayed less regular boundaries, in comparison to untreated MSC morphology (Figure 1E). Despite these morphology changes, we never revealed, even in long-term culture (up to 8 weeks), the presence of uncontrolled expanding clones following stress induction.

**Figure 2:**
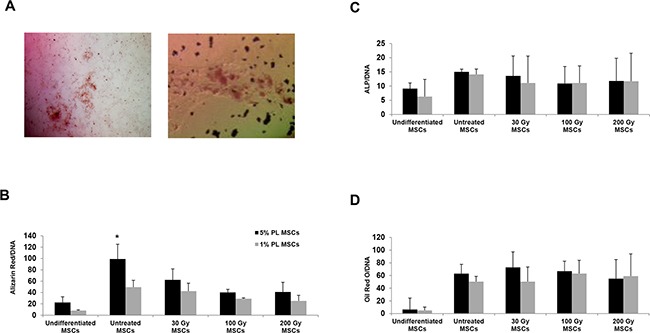
Histological staining and quantification of the osteogenic and adipogenic differentiation capacity of huMSCs isolated from one representative sample before and after irradiation and nutrient deprivation **A.** Histological staining with Alizarin Red (for osteoblasts, left panel) and Oil Red O (for adipocytes, right panel). **B, C.** quantification of osteogenic differentiation capacity by evaluating the deposits of calcium (positivity for the Alizarin Red staining, B) and the alkaline phosphatase activity (C). **D.** quantification of the differentiation into adipocytes by assessing the amount of Oil Red O stained lipid droplets. The amount of dye is related to the amount of DNA extracted from the same wells and is expressed in mOD/μg. Each bar represents the mean +/−SD of three experiments. * stands for P<0.05 calculated for the comparison between untreated and irradiated 5% PL MSCs.

In order to compare the differentiation potential of stressed MSCs with that of untreated MSCs, we induced MSC differentiation into osteoblasts and adipocytes and evaluated results by histological staining. At any IR doses, both irradiated and starved MSCs retained their adipocytic differentiation potential, as confirmed by Oil Red O positive staining of lipid droplets in cell cultures (Figure 2A, right panel and 2D). Although differentiating into osteoblasts, irradiated MSCs showed a significantly lower ability to form calcium deposits as compared with untreated 5% PL-MSCs (P<0.05 for all IR doses), as shown by Alizarin Red quantization (see Figure 2B). No significant differences were observed between untreated and stressed MSCs when cells were cultured with 1% PL.

### IR and starvation culture effect on cell cycle progression and cell viability

**Figure 3:**
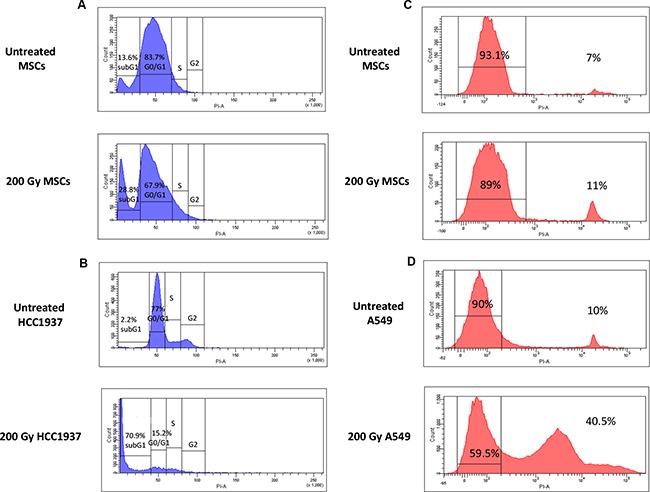
Cell-cycle and cell-viability analysis of untreated and 200 Gy irradiated and starved MSCs Left panel **A**, **B.** the picture shows representative FACS analysis of untreated and stressed MSCs (in the presence of 1%PL). As indicated by cell-cycle hystograms, after induction of stress, most MSCs remain quiescent in G0/G1 phase (A), whereas the totality of HCC1937 cells, after stress, becomes apoptotic (subG1 phase, B). Right panel **C**, **D.** cell viability assay indicates a minor percentage of dead cells after induction of stress for MSCs (11%, C), whereas a higher percentage of dead cells among radio-resistant A549 cells is shown (41.5%, D). Experiments were conducted in triplicate for each condition.

The effect of ionizing radiation and serum deprivation on MSC cell-cycle progression was evaluated after stress induction. The fraction of untreated MSCs in G0/G1 phase of the cell cycle (during which cells are in a quiescent state and are committed to enter cell cycle) was 83.7%, whereas the fraction of cells in subG1 phase (where apoptotic cells are situated) was 13.6%. As shown in Figure 3A, after IR treatment and serum deprivation for 21 days, the fraction of G0/G1 cells decreased up to 67.9% and the subG1 population raised to 28,8% in 200-Gy irradiated and starved MSCs. Conversely, after the same treatment, the radio-sensible cell line HCC1937 showed a marked reduction in G0/G1 cycle progression (15.2%) and a consequent increase in subG1 population (up to 70.9%; Figure 3B), this confirming the higher resistance of MSCs to IR and starvation induced apoptosis.

To investigate whether physical/chemical stressors could affect MSC viability, cells were stained with propidium iodide and their viability was measured by flow-cytometry and compared with that of the radio-resistant A549 cell line. As shown in Figure 3C-D, MSC cell viability decreased from 93.1% (untreated MSCs) to 89% (200 Gy/starved MSCs), whereas A549 cell viability decreased from 90% (untreated A549) to 59.5% (200 Gy starved A549), thus showing a superior resistance of MSCs to irradiation-induced apoptosis as compared with a known radio-resistant cell line.

### Effect of irradiation and starvation culture on MSC immunomodulatory capacity

**Figure 4:**
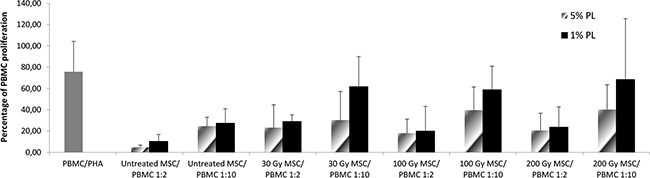
*In vitro* immunomodulatory effect of untreated and stressed MSCs on PBMC proliferation The graph shows the proliferation of healthy donor PBMCs stimulated with phytohemagglutinin (PHA) either in normal conditions or in starvation conditions. Each bar represents the percentage of proliferation of 10^5^ PBMCs, in the presence of two different MSC:PBMC ratios (MSC:PBMC ratio of 1:2 and 1:10), calculated by measuring 3H-thymidine incorporation after 3-day co-culture. Grey bars refer to 5% PL-cultured MSCs, whereas black bars refer to 1% PL- cultured MSCs. The count per minute (cpm) values at each cell concentration were normalized to the cpm of PBMCs without MSCs in each experiment. Each bar represents the mean ± SD of five experiments (each point being in triplicate) with MSCs obtained from 5 HDs.

In order to evaluate whether exposure to IR and/or starvation culture conditions could affect the immunomodulatory capacity of MSCs, we measured the percentage of proliferation of peripheral blood mononuclear cells (PBMCs) after stimulation with phytohemagglutinin (PHA) either in the presence or in the absence of stressed MSCs in an allogeneic setting. While in the absence of MSCs, PBMC proliferation to PHA was 75.80% (SD ± 28.5), as previously reported [24, 25], a marked *in vitro* inhibitory effect exerted by untreated MSCs on the proliferation of PHA-stimulated PBMCs was demonstrated: mean percentage of proliferation 4.70% (SD ± 2.11; P<0.001 in comparison to PHA+PBMCs alone) and 24.58% (SD ± 8.37; P=0.004 in comparison to PHA+PBMCs alone) at MSC:PBMC ratios of 1:2 and 1:10, respectively. MSC capacity to inhibit allogeneic PBMC proliferation was unaltered after 30 Gy-irradiation: PBMC mean proliferation in the presence of 30 Gy-irradiated MSCs was 23.23% (SD ± 21.37; P=0.01 in comparison to PHA+PBMCs alone) and 30.35% (SD ± 26.86; P=0.03 in comparison to PHA+PBMCs alone) at ratios 1:2 and 1:10, respectively (Figure 4). Superimposable results were achieved when MSCs irradiated at higher doses (100 and 200 Gy) were added to the co-culture (see Figure 4). Therefore, at any IR dose, the inhibitory effect exerted by MSCs on PBMC proliferation was maintained and was dependent on MSC:PBMC ratio, suggesting that IR does not seem to alter *in vitro* MSC immune-regulatory capacity. When PBMCs were co-cultured with irradiated and starved MSCs, no statistically significant difference between untreated and stressed MSCs was revealed at 1:2 MSC:PBMC ratio, whereas the inhibitory effect of irradiated and starved MSCs was less evident at 1:10 MSC:PBMC ratio (Figure 4).

These data were confirmed by the analysis of soluble factors and cytokines in culture supernatants after PBMC co-culture. As far as anti-inflammatory cytokines are concerned, the co-culture of PBMCs with irradiated and starved MSCs resulted in the same secretion pattern of the co-culture with untreated huMSCs. In particular, no statistically significant difference in IL6, IL10 and TGFβ levels was revealed in co-cultures with stressed MSCs, in comparison with the conditions PHA+PBMCs+untreated MSCs for both MSC:PBMC ratios (see Supplementary Figure S1A and S1B). Measurements of the pro-inflammatory cytokine IFNγ and of some soluble factors known to mediate MSC immunomodulating effect (i.e. HGF and Galectin-1), were not significantly different in supernatants collected from co-cultures of PHA-stimulated PBMCs and stressed MSCs and in supernatants collected from PHA+PBMCs+untreated MSCs co-cultures. In detail, IFNγ levels diminished also in the presence of stressed MSCs, whereas HGF and Galectin-1 levels increased after exposure to stressed MSCs (see Supplementary Figure S2A and S2B). Cytokine and soluble factor levels measured in the presence of MSCs exposed to 100 and 200 Gy IR showed superimposable results (data not shown).

### Genetic characterization of stressed MSCs

To assess the potential risk of transformation into malignant cells following stress exposure, despite phenomena of MSC outgrowth failed to occur, MSC genetic profile was characterized by means of array-CGH and conventional karyotyping after IR and starvation culture.

**Figure 5:**
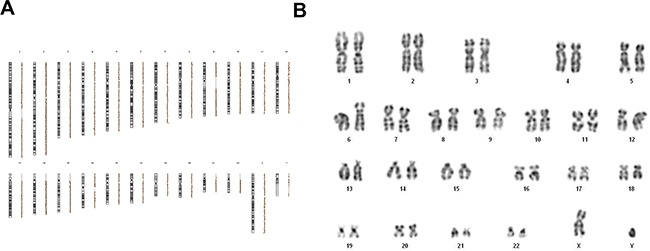
Array-CGH profile **A.** and cytogenetic analysis through conventional karyotype **B.** of one representative MSC sample irradiated at 200 Gy and cultured with nutrients deprivation. Both array-CGH and karyotype were performed on all 20 HD-MSCs. The profiles are linear; neither unbalanced chromosomal rearrangements nor chromosomal aberrations were shown.

Array-CGH analyses of irradiated and starved MSCs did not reveal any imbalanced chromosomal alteration. Indeed, as shown in Figure 5A, no DNA deletion or duplication at any irradiating dose (including the condition of irradiation with 200 Gy) was detected. Since balanced genetic aberrations cannot be detected by array-CGH, in order to better define the genetic profile of stressed MSCs, the same MSC samples investigated by array-CGH were analyzed through conventional karyotyping. By this latter assay, chromosomal alterations were not detected in MSCs after exposure to IR and starvation culture (Figure 5B). It has to be pointed out that, since at very high IR doses it was not possible to collect enough metaphases (≥20), karyotyping was performed only on 30 Gy-irradiated MSCs.

Conversely, the genetic characterization by conventional karyotyping of the two control cell lines (*i.e.* A549 and HCC1937) showed many chromosomal anomalies and double strand breaks after IR treatment [26], even at the lowest irradiation dose of 30 Gy. Considering the polyploidy of both cell lines and their naturally occurring chromosomal rearrangements, we analyzed their genetic profile at the first passage in culture, after four passages and after IR treatment. Both A549 (although known to be radio-resistant at 16Gy) and HCC1937 (radio-sensitive) [27, 28] did not accumulate chromosomal rearrangements after passaging in culture; however, they showed substantial chromosomal breakings already after 30 Gy IR exposure (see Supplementary Figure S3; at higher IR doses and serum deprivation conditions, genetic analysis was not performed due to massive cell death and consequent no availability of sufficient metaphases).

Taken together, these results demonstrate that MSCs are not prone to develop neoplastic transformation after exposure to supramaximal physical and chemical stressors. Neither IR, reported to obstacle DNA synthesis and to induce DNA damage, nor nutrients deprivation, often present in the tumor hypoxic microenvironment, could induce MSC genetic alterations.

### Activation of stress-induced DNA damage response pathway

**Figure 6:**
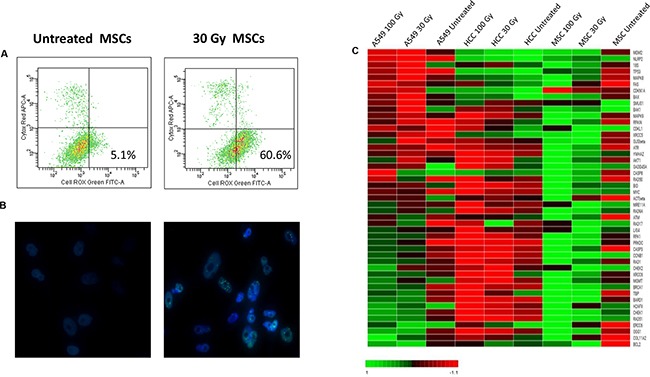
Analysis of ROS level and DDR pathway activation after induction of stress In **A.** ROS levels were measured by flow-cytometry in untreated and 30 Gy irradiated MSCs in the presence of 5%PL. In **B.** the analysis of DNA-damage induced ATM phosphorylation of one representative sample is shown. Both untreated and 200 Gy irradiated MSCs were stained using an anti-phosphoATM primary antibody and a FITC-labeled secondary anti-rabbit IgG antibody. DNA was stained using DAPI. Images were captured using a Leica DMI8 fluorescence microscope. In **C.** a heatmap representation of gene expression analysis performed on untreated and stressed MSCs, A549 and HCC1937 is shown. 200 Gy irradiated condition is not represented since RNA quality after isolation was not satisfactory. Data shown are representative of one MSC donor; similar analyses were performed on 5/20 HD-MSCs with superimposable results.

Besides cell-cycle arrest with early replicative senescence and eventually cell death, exposure to IR is known to cause the production of intracellular reactive oxygen species (ROS) and to induce single- and double-strand DNA breaks [29]. In our experimental setting, IR treatment induced the increase of ROS-producing cells up to 75% (after 200 Gy irradiation and starvation culture), resulting in cell injury and DNA damage (see Figure 6A). Since it has been shown that a network of damage sensors, signal transducer and repair effector molecules mediates the response of cells to DNA damage [30], by means of immunofluorescence and gene expression analysis, we characterized the effects of γ-irradiation and starvation culture on a number of key DNA damage response pathways in MSCs and in two control cell lines (A549 and HCC1937, a radio-resistant and a radio-sensitive cell line, respectively). By immunofluorescence, we observed the phosphorylation of intracellular ataxia telangiectasia mutated protein (ATM), a crucial factor responsible for the recruitment and activation of multiple downstream DNA damage sensing and repair proteins in stressed MSCs (Figure 6B). Noteworthy, immunofluorescent staining of stressed MSCs showed an increase in the number of pATM foci with escalating doses of IR, whereas this effect was less evident in irradiated and starved MSCs (data not shown). In order to evaluate the role played by DDR pathway in MSC resistance to malignant transformation, we performed gene expression analysis on cDNA samples isolated from untreated and stressed MSCs and from the two control cell lines A549 and HCC1937. As shown in Figure 6C, the expression of genes known to be responsible for the non-homologous end-joining (NHEJ) repair of double strand breaks (DSBs), such as PRKDC, Rad50, H2AFX and ATM increased in MSCs in response to increasing doses of IR. Similarly, proteins responsible for the activation of homologous recombination (HR) pathway, such as RPA1, BRCA1 and Rad51, showed a considerable higher expression in MSCs after the induction of physical and chemical stress. Moreover, as demonstrated by cell-cycle progression analysis, after IR and starvation treatment, most MSCs stopped at G1 checkpoint, at which cells are ready to enter the cell cycle, but did not proceed further this restriction point. The decision to undergo a new round of cell division normally occurs when cyclin-CDK-dependent transcription process is activated, followed by the entry into S phase; interestingly, cyclin-CDK-dependent CDKN1a gene (coding for p21 protein), constitutively expressed in untreated MSCs, became downregulated after stress induction, whereas other DNA damage sensors involved in cell cycle arrest, such as p53, CHEK1 and CHEK2, were found to be more expressed in stressed cells, thus confirming the blockade of cell proliferation after IR treatment. Finally, genes responsible for cell survival/apoptosis switch, *i.e.* BAK, BCL 2, CASPASE 3 and 8 and COL11A2 (coding for PARP), resulted to be overexpressed after IR and starvation culture. Similar gene expression patterns were found in MSCs irradiated in the presence of 1%PL, although at a less extent than 5%PL cultured cells, this suggesting a minor role played by DDR pathway in nutrient deprivation conditions (data not shown). As for control cell lines, in radio-resistant A549 cells a higher expression of DDR responsible genes was observed after IR treatment with increasing expression in response to escalating IR doses, although at a lower extent than stressed MSCs (see Figure 6C). Conversely, there was hardly any DDR activation in the stressed radio-sensible HCC1937 cells.

These data confirm huMSC resistance to IR and starvation culture, which may be, at least partly, mediated by the activation of DDR pathways in response to stressors.

## DISCUSSION

The present study provides support to the biosafety profile of *ex-vivo* expanded huMSCs even after exposure to supramaximal stress by demonstrating that, despite altered morphology and growth rate, MSCs are resistant to IR and starvation culture and do not display propensity to transform into malignant cells.

Two previous studies have shown that the biological and functional profiles of AT- and BM-derived MSCs could be altered after long-term *in vitro* culture, which promoted the onset of genetic alterations leading to neoplastic transformation [16, 17, 18, 19]. These reports have been subsequently retracted and re-interpreted in light of the contamination of the MSC cultures by tumor cell lines [32, 33]. Moreover, other researchers, including our group, have shown that long-term *in vitro* culture of huMSCs can be performed without altering their typical phenotypical/functional profile and without inducing genetic aberrations [22, 31]. To better delineate the genetic profile of BM-derived huMSCs, we employed both conventional karyotyping and array-CGH and documented the absence of genetic abnormalities in these cells after long-term expansion [22, 31]. Altogether, these data suggest that malignant transformation is likely to be an uncommon event in huMSCs *ex vivo* expanded in standard, plastic-adherent culture conditions and in the presence of foetal bovine serum [19].

Starting from this notion, our initial experimental plan was to artificially induce, through high-dose IR and starvation culture, malignant transformation in our MSC culture system, which was based on an animal-free, PL-based culture medium. This would have had potentially allowed us to generate a transformed huMSC population to be characterized and studied, in order to get insight into mechanisms of malignant transformation in *ex-vivo* cultured huMSCs. Despite the application of potent supramaximal stress, we were not able to induce malignant transformation in our cells, this confirming that huMSCs are not prone to undergo neoplastic changes also after application of physical/chemical stressors and under *ex-vivo* culture conditions, which include PL that, due to the high content in platelet-derived growth-factors, has been reported to induce an intense proliferative drive on *ex-vivo* cultured MSCs [34]. Exposure to IR is known to significantly influence viability and proliferative capacity of irradiated cells; however, MSCs have been shown to be relatively radio-resistant as compared with other BM-derived stem cells when exposed to a maximal dose of 12 Gy [35]. To obtain a combined physical-chemical stressful environment, we have also employed nutrient deprivation, which is known to occur during disease processes, such as ischemia and infection [36, 37]. While the precise mechanisms underlying MSC distress due to serum deprivation remain to be elucidated, it has been reported that processes related to caspase-induced apoptosis pathway, such as loss of nuclear and membrane integrity, chromatin condensation and reduction in cell size can be triggered even by a brief (*i.e.* 24 hours-treatment) nutrient deprivation. Moreover, re-feeding starved cells with growth factors or serum has been shown to induce powerful proliferative drive [38, 39]; indeed, in our protocol, it was employed to induce further cellular stress. Following exposure to this combination of stressing agents, we performed a thorough phenotypical, functional and genetic characterization of MSCs isolated from BM of 20 HDs. Our data demonstrate that, with escalating IR doses (up to 200 Gy), MSCs proliferative capacity becomes significantly lower in comparison to untreated MSCs; these results are in line with a previously reported study employing 12 Gy irradiation [40]. Despite this reduced proliferation rate, irradiated MSCs were still capable of adhering to plastic surface and displayed the typical MSC surface markers, this suggesting that MSC adhesion properties and phenotype are not affected by IR exposure. These observations were confirmed when, after IR treatment, MSCs were also cultured in starvation conditions. The noticed morphological changes associated with IR exposure were less evident when MSCs were cultured with nutrient deprivation. This latter finding might be explained by the reduced proliferative pressure induced by the lower concentration of platelet-derived growth factors in the starvation culture conditions. As regards cell cycle analysis, our results confirm the blockade of cell cycle progression induced in MSCs by IR and serum deprivation, without triggering apoptosis and cell death. Moreover, no statistically significant difference was observed between untreated and irradiated MSCs in their immunomodulatory effect; only after starvation/re-feeding culture, a reduction in MSC inhibitory capacity was observed at 1:10 MSC:PBMC ratio, this possibly reflecting the exhaustion of some primitive MSC functions when the level of stress exceeds a certain limit. A similar observation can be made when considering the reduced ability to form Alizarin Red-positive calcium deposits in irradiated and starved MSCs, especially at the higher IR doses. The genetic characterization indicates that MSCs are not prone to undergo transformation after exposure to IR; moreover, also the lack of nutrients followed by re-feeding did not seem to trigger the development of genetic abnormalities. Our data indicate that MSC radio-resistance, already reported at much lower IR doses [41, 42], seems to be maintained at supramaximal doses of radiation and after starvation culture.

In order to investigate the mechanisms of MSC resistance to supramaximal stress, we evaluated some of fundamental mechanisms underpinning cell defense against radiation and, in particular, the role of DDR pathway [30]. Since IR exerts its effects mostly on cell genomic information, either by directly depositing its energy onto DNA molecules or by creating free radicals that, in turn, interact with the DNA strands, we first investigated and demonstrated the intracellular activation of ATM [43], a central regulatory protein of DDR pathway, which binds to sites of DNA breaks and is responsible for phosphorylation of various downstream components, including Chk2 protein and the histone variant H2AX. Noteworthy, the level of ATM activation, measured by counting pATM foci, increased in response to escalating IR doses. While base damage or single-strand breaks occur much more frequently, DNA double strand breaks are considered the main toxic lesion by which IR kills cells. Swift recognition and repair of DNA damage is crucial for affected cells; failure to repair may result in cell death and misrepairing may lead to an accumulation of mutations and genomic instability. Based on these considerations, we decided to perform an in-depth evaluation of the mechanisms responsible for MSC resistance to stressors and consequent maintenance of genomic stability. Gene expression analysis on stressed MSCs indicated a strong induction of NHEJ-related proteins (such as Rad51, ATM, H2AFX, XRCC5 and ERCC6), as well as the activation of HR pathway-dependent molecules (such as RPA1 and BRCA1) in response to high IR doses. Additionally, we demonstrated that IR-induced damage can trigger the activation of the DNA damage response, as shown by the activation of p53, that subsequently promote the delay or even the arrest of cell-cycle, in order to allow DNA repair before the cell cycle progresses [44, 45]. Moreover, as previously described [46], our study indicates that MSC radio-resistance is at least partly mediated by the equilibrium between the levels of pro-apoptotic and anti-apoptotic proteins (as shown by the strong induction of BCL2 and MYC).

In conclusion, our study suggests that exposure to massive doses of IR and deprivation of nutrients affects MSC morphology and proliferation rate by inducing early replicative senescence; however, it does not alter the phenotypical and functional properties of MSCs and it does not influence their genetic stability. Moreover, we showed that the activation of DDR pathway in MSCs undergoing supramaximal stress may contribute to prevent their oncogenic transformation. In view of the broader use of MSCs in the clinical arena, our data indicate that low-dose irradiation might be employed to abolish MSC proliferation before infusion, without compromising their immunomodulatory activity. Finally, data obtained through our *in vitro* stress model may be employed by Regulatory Authorities with the aim to support the safe employment of *ex vivo* expanded huMSCs in PL-supplemented medium and may serve as a prototype system to study stem cell aging in stressing conditions.

## MATERIALS AND METHODS

### MSC isolation and ex-vivo expansion

After obtaining written informed consent, MSCs were isolated from residual cells of 20 HDs (median age 16 years, range 5-32) who donated BM for hematopoietic stem cell transplantation at Bambino Gesù Children's Hospital, Rome, Italy. Mononuclear cells were isolated by density gradient centrifugation (Ficoll 1.077 g/ml; Lympholyte, Cedarlane Laboratories Ltd., The Netherlands) and plated in non-coated 75-175 cm^2^ tissue culture flasks (BD Falcon, NJ, USA) at a density of 160000/cm^2^ in complete culture medium: DMEM (Euroclone, Milan, Italy) supplemented with 5% PL, 50 U/ml penicillin, 50 mg/ml streptomycin and 2 mM L-glutamine (Euroclone). PL was prepared as previously described [34]; expired platelet apheresis were obtained from 10 healthy volunteers at the Transfusion Service of our Hospital and were previously screened for infectious agents according to Italian legislation. PL preparations obtained from the different donors were pooled in a single culture supplement to be used for the generation and expansion of MSCs. Cultures were maintained at 37°C in a humidified atmosphere, containing 5% CO_2_. After 48-hour adhesion, non-adherent cells were removed and culture proceeded with culture medium being replaced twice a week. MSCs were harvested, after reaching ≥80% confluence, using Trypsin (Euroclone), and were propagated at 4000 cells/cm^2^ up to P2.

### Irradiation and nutrient starvation

Once they had reached P2 in culture, MSCs were detached and exposed to escalating doses of γ-rays (30, 100 and 200 Gy) by means of Gamma Cell 1000 Elite irradiator (Nordion International, CDN). Following irradiation, nine out of the 20 MSC samples were also cultured in the presence of 1% PL-supplemented medium at 4000 cells/cm^2^. In this second cohort of MSC samples, at each passage, 1% PL- and 5% PL- supplemented media were alternated, in order to stress cells in starvation/refeeding culture conditions. After trypsinization, cells were harvested and characterized for proliferative capacity, phenotype, functional properties and genetic profile and compared with untreated MSCs. Cultures were maintained at 37°C in a humidified atmosphere, containing 5% CO_2_, and after expansion they were exposed to escalating doses of γ–rays (30, 100 and 200 Gy) and then to serum deprivation culture, as previously described for MSCs. A549 cell line (a human hypotriploid Non-Small Cell Lung Carcinoma, NSCLC, cell line) and HCC1937 (a primary ductal breast carcinoma cell line with a modal number of 100 chromosomes) were obtained from ATCC (LGC Standards, Middlesex, UK) and cultured following manufacturer's instructions; cell lines were employed as radio-resistant and radio-sensitive control cell line, respectively, and were submitted to the same treatment of MSCs.

### Characterization of stressed MSCs

#### Proliferative capacity

Cell growth was analyzed by direct cell counts, and population doublings (PDs) were determined at each passage. The number of PDs was calculated for each MSC sample by using the formula log10(N)/log10(2) where N means cells harvested/cells seeded; results were expressed as cumulative PDs from passage P1 to P6 [47].

#### Immune-phenotype

MSCs were phenotypically characterized by means of direct immunofluorescence with a FACSCanto flow-cytometer (BD PharMingen, San Diego, CA). Fluorescein isothiocyanate (FITC)- or phycoerythrin (PE)-conjugated monoclonal antibodies specific for CD13, CD14, CD34, CD45, CD73, CD80, CD90, class I-HLA and HLA-DR, CD73, CD105 (BD PharMingen) were used. Appropriate, isotype-matched, non-reactive fluorochrome-conjugated antibodies were employed as controls. Analysis of cell populations was performed and data were calculated using the FACSDiva software (Tree Star, Inc. Ashland, OR).

#### Senescence assay

After exposure to IR and starvation, senescence of MSCs was evaluated by direct-light microscopy and by staining with a senescence β-galactosidase (SA-β-gal) Staining Kit (Cell Signaling Technology, Danvers, MA), according to manufacturer's instructions.

#### Cell cycle and viability analyses

For cell cycle, after IR and starvation culture, cells were fixed in ice-cold 70% ethanol and incubated for 30 min at 37°C with a PBS solution containing 20 μg/ml propidium iodide and 100 μg/ml RNAse (BD Biosciences). Cell-cycle progression was analyzed by flow-cytometry using a FACSCanto flow-cytometer (BD PharMingen). Cell viability was assessed by staining cells with 10 μg/ml Propidium Iodide Solution (BD Biosciences). Both assays were analyzed using the FACSDiva software (BD PharMingen).

#### Differentiation capacity

The osteogenic differentiation capacity of MSCs was assessed at P3 both for stressed and untreated MSCs by incubating cells with αMEM (Euroclone), 5% PL, 50 U/ml penicillin, 50 mg/ml streptomycin, and 2 mM L-glutamine supplemented with 10-7M dexamethasone and 50 mg/ml L-ascorbic acid; starting from day +7 of the culture, 5 mM ß-glycerol phosphate (Sigma-Aldrich, St Louis, MO) was added to the medium. Adipogenic differentiation was evaluated at P3 both for stressed and untreated MSCs by incubating cells with αMEM, 5% PL, 50 U/ml penicillin, 50 mg/ml streptomycin, and 2 mM L-glutamine supplemented with 10-7M dexamethasone, 50 mg/ml L-ascorbic acid, 100 mg/ml insulin, 50 mM isobutyl methylxanthine, 0,5 mM indomethacin (Sigma-Aldrich) and 5 mM b-glycerol phosphate. Both osteogenic and adipogenic cultures were incubated for at least two weeks before differentiation was evaluated. To detect osteogenic differentiation, cells were stained for alkaline phosphatase (AP) activity using Fast Blue (Sigma-Aldrich) and for calcium deposition with Alizarin Red (Sigma-Aldrich). Adipogenic differentiation was evaluated through the morphological appearance of fat droplets stained with Oil Red O (Sigma-Aldrich). AP, Alizalin Red and Oil Red O extraction were measured spectrophotometrically at 550nm and compared to a standard titration curve for quantification of osteogenic and adipogenic differentiation, as previously described [48].

#### PBMC proliferation assay with PHA

PBMCs were obtained by conventional Ficoll separation from heparinized peripheral blood samples from 10 HDs who gave informed consent for participation to this study; cells were employed on the same day of collection. The proliferation of PBMCs in RPMI 1640 medium (Gibco, Life Technologies Ltd, CA) supplemented with 10% FBS, in response to phytohemagglutinin (PHA-P; Sigma-Aldrich), either in the presence or absence of BM-derived MSCs, before and after exposure to physical/chemical stressors, was performed in triplicate in flat-bottom 96-well tissue culture plates (BD Falcon, Franklin Lakes, NJ). Briefly, MSCs were seeded at MSC:PBMC ratios of 1:2 and 1:10 per well and allowed to adhere overnight before adding 10^5^ PBMCs per well with or without PHA (5 μg/ml). After 3-day incubation at 37°C in a humidified 5% CO_2_ atmosphere, cultures were pulsed with ^3^H-thymidine (1 μCi/well, specific activity 6.7 Ci/mmol, Perkin Elmer, Waltham, MA) and harvested after 18 hours. ^3^H-thymidine incorporation was measured by standard procedure with Microbeta Trilux 1450 instrument (Perkin Elmer); results were expressed as percentage of proliferation. All the experiments were performed in an allogeneic setting.

#### Genetic profile of stressed MSCs

Molecular karyotyping on stressed MSCs was performed through array-comparative genomic hybridization (array-CGH) with the Agilent kit (Human Genome CGH Microarray, Agilent Technologies, Santa Clara, CA) on each of the 20 HDs. The array-CGH platform is a 60-mer oligonucleotide-based microarray that allows a genome-wide survey and molecular profiling of genomic aberrations with a resolution of about 41 kb (kit 60K). DNA was extracted from MSCs with QIAamp^®^ DNA Blood Kit (QIAGEN) according to the manufacturer's instructions. DNA (1 μg) from stressed MSCs and unstressed MSCs, as controls, of the same sex (control DNA, Promega, Madison, WI) were processed according to the reported protocol (Agilent Oligonucleotide Array-Based CGH for Genomic DNA Analysis – Version 6.2.1, February 2010). The array was analyzed through the Agilent Scanner and the Feature Extraction software (v10.7.3.1) and Agilent Genomic Workbench Lite Edition 6.5.0.18.

Stressed MSCs were also analyzed by conventional karyotyping. Before harvesting, MSCs at P6 were incubated at 37°C with colcemid (IrvineScientific, Santa Ana, CA) at 1 μg/ml final concentration for 5 hours. Then, cells were fixed and spread according to standard procedures. Metaphases of cells were GTG-banded and karyotyped in accordance with the International System for Human Cytogenetic Nomenclature recommendations (ISCN, 2009). At least 20 metaphases were analyzed for each sample.

Conventional karyotyping was also performed on the control cell lines (A549 and HCC1937) before and after irradiation at 30 Gy.

### Analysis of DNA damage response pathway

#### Gene expression analysis

The effects of γ-irradiation and starvation culture on gene expression of MSCs (three different samples) and two control cell lines (A549 and HCC1937) were assessed using custom Taqman^®^ array 96-well Fast plates (Applied Biosystems, Life Technologies), previously designed to measure the expression level of genes involved in DDR pathway and cell cycle (47 target genes plus 1 endogenous control, in duplicate). Briefly, after irradiation and starvation culture, RNA was isolated with TRIzol (Life Technologies) followed by phenol-chloroform extraction and reverse transcription. Then, 25 ng of cDNA per well of each sample were plated in custom array plates (each well corresponding to one gene assay) and quantitative real-time PCR was performed by means of Quantstudio 12K Flex instrument (Life Technologies). Data were analyzed with Quantstudio Analysis software (Applied Biosystem). Statistical analysis was performed using paired Student's t-test.

#### Immunofluorescence staining

Both untreated and irradiated/starved MSCs were plated on culture slides (BD Falcon). After fixation in 4% paraformaldehyde for 20 minutes, permeabilization with 0.1% SDS for 10 minutes and blocking with 3% bovine serum albumin for 20 minutes, cells were incubated with anti-ATM rabbit monoclonal antibody (phospho S1981; Abcam, Cambridge, UK) for 14 hours at 4°C followed by incubation with goat anti-rabbit polyclonal Alexa Fluor 488 antibody (Abcam) for 1 hour at 37°C. Nuclei were subsequently stained with Hoechst nucleic acid stain (Molecular Probes, Life Technologies). Cells were finally mounted with 3% glycerol solution and observed under a fluorescence microscope (Leica DMI8) with 40x magnification. The number of phospho-ATM foci was determined using ImageJ software.

#### Detection of ROS by flow-cytometry

ROS levels were measured by CellROX^®^ Green Flow Cytometry assay kit (Molecular Probes, Life Technologies Ltd). Briefly, after IR and starvation culture, cells were incubated for 30 minutes at 37°C with 1μm CellROX^®^ Green reagent and with 5μm of SYTOX^®^ Red Dead Cell Stain. Then, samples were analyzed by flow-cytometry, using 488-nm excitation for the CellROX^®^ Green reagent and 639-nm excitation for the SYTOX^®^ Red stain at FACSCanto flow-cytometer (BD PharMingen); results were analyzed with FACSDiva Software.

### Statistical analysis

Comparisons on the equality of means of PDs were performed by using the Student's t-test, assuming paired data. The two-sample test of proportion was used to compare data expressed in percentages (PHA-induced PBMC proliferation). For both tests, a p-value lower than 0.05 was considered to be significant. Statistical analysis was performed using the Stata/IC 11.0 software package. Data were analyzed in April, 2016.

## SUPPLEMENTARY MATERIALS METHODS AND FIGURES


