# Supplementary material for: Resistance to neoplastic transformation of ex-vivo expanded human mesenchymal stromal cells after exposure to supramaximal physical and chemical stress
Source: Oncotarget. 2016 Oct 15;7(47):77416–29. doi: 10.18632/oncotarget.12678 (PMC5363595; doi:10.18632/oncotarget.12678)
Supplement: Supplementary file 1 [file oncotarget-07-77416-s001.pdf]

Resistance to neoplastic transformation of *ex-vivo* expanded human mesenchymal stromal cells after exposure to supramaximal physical and chemical stress

SUPPLEMENTARY MATERIALS AND METHODS

Measurement of growth factors and cytokines

The concentration of IL-6, IL-10, TGFβ and IFNγ in supernatants of co-cultures of MSCs (both untreated and irradiated/starved) with PBMCs/PHA, after 72 hours incubation, was quantified by means of commercially available ELISA kits obtained from Mabtech (Nacka Strand, Sweden). The analysis of HGF and Galectin-1 content in the same supernatants was performed using ELISA kits obtained from R&D System (Minneapolis, MN, USA), following the manufacturer's instructions. Plates were read either at 405 nm (for ALP-conjugated antibodies) or at 450 nm (for HRP-conjugated antibodies) through Envision Multilabel Reader (Perkin Elmer).

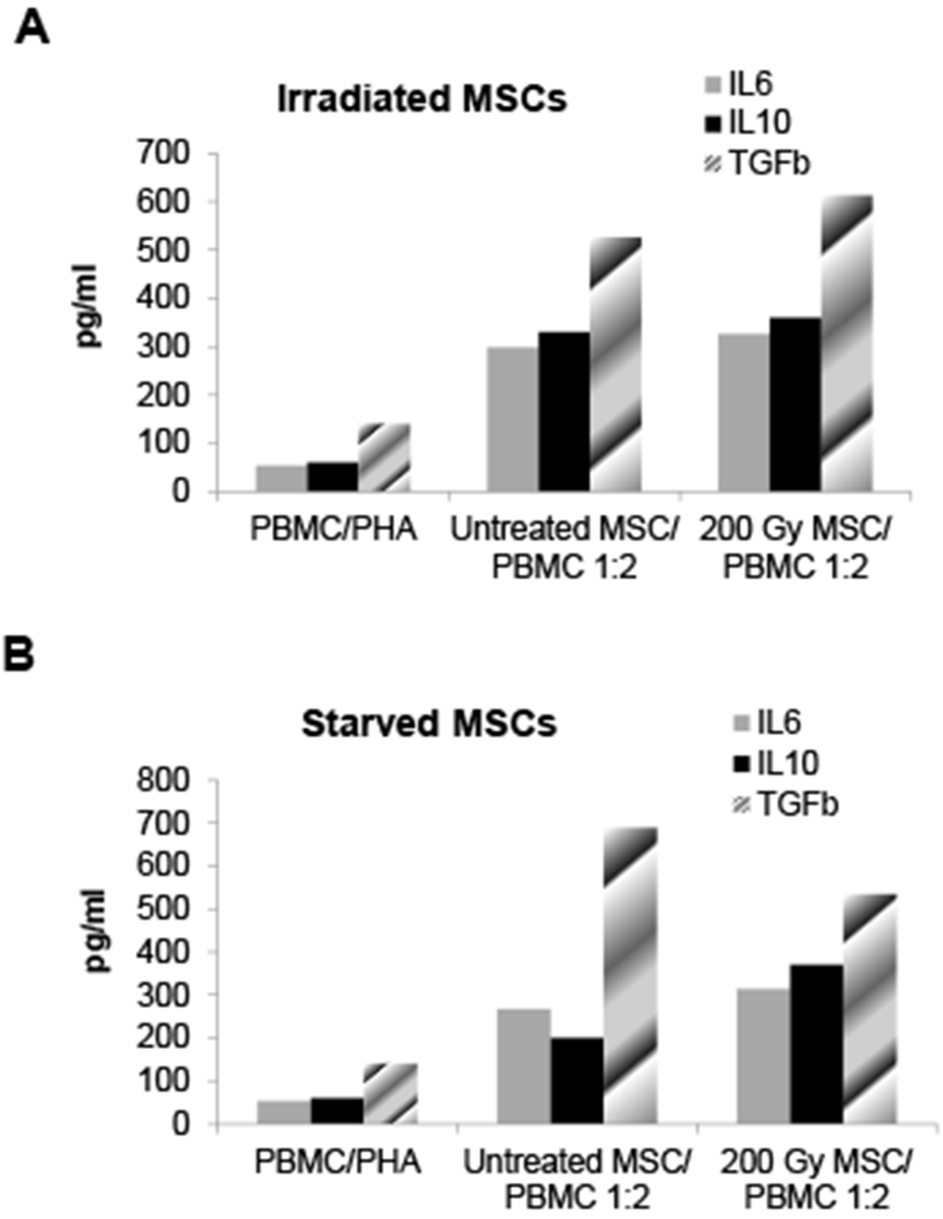

Supplementary Figure S1: Concentrations of anti-inflammatory cytokines in supernatants of co-culture of PHA stimulated PBMCs with either untreated or stressed MSCs. Quantification was performed by ELISA; one representative experiment is shown. Results are expressed as pg/mL. Measurement of the inhibitory/anti-inflammatory cytokines IL6 and IL10 and growth factor TGFβ in co-cultures of PHA-stimulated PBMCs with irradiated MSCs **A.** or starved MSCs **B.**

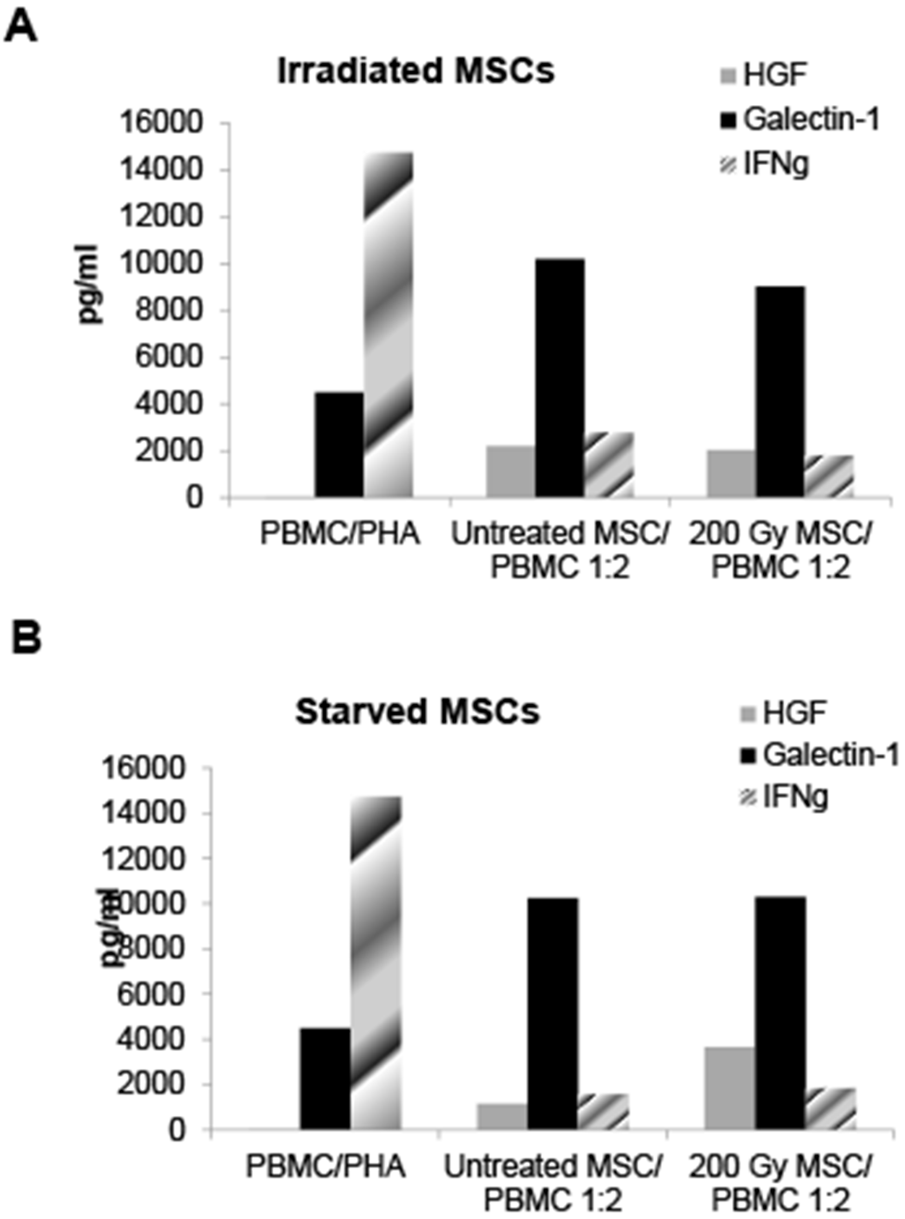

Supplementary Figure S2: Concentrations of pro-inflammatory cytokines and growth factors in supernatants of co-culture of PHA stimulated PBMCs with either untreated or stressed MSCs. Quantification was performed by ELISA; one representative experiment is shown. Results are expressed as pg/mL. Measurement of the stimulatory/pro-inflammatory cytokines IFNγ and of paracrine factors HGF and Galectin-1 in co-cultures of PHA-stimulated PBMCs with irradiated MSCs **A.** or starved MSCs **B.**

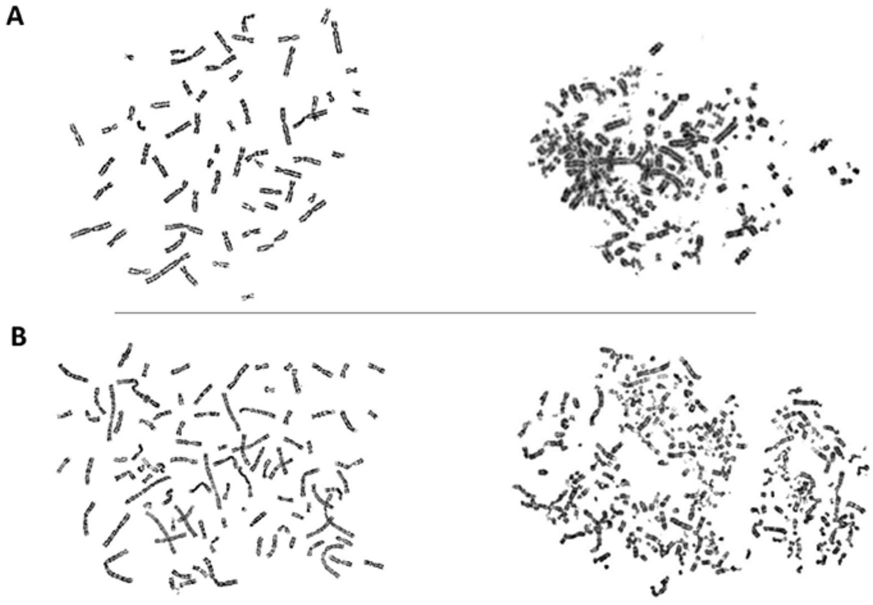

Supplementary Figure S3: Cytogenetic analysis, performed through conventional karyotype, of A549 (A) and HCC1937 (B). cell lines, after four passages in culture, before (on the left) and after (on the right) 30Gy IR treatment. The pictures indicate that IR treatment, even at the lowest dose, is able to induce many DNA breaks in both control cell lines.
